# Mindfulness meditation as a tool for enhancing recovery and reducing burnout in elite tennis players

**DOI:** 10.3389/fpsyg.2025.1661724

**Published:** 2025-10-23

**Authors:** Min Liao

**Affiliations:** ^1^College of Physical Education, Sichuan Technology and Business University, Chengdu, Sichuan, China; ^2^Department of Education, Faculty of Social Sciences and Liberal Arts, UCSI University, Kuala Lumpur, Malaysia

**Keywords:** mindfulness meditation, psychological recovery, burnout, psychological resilience, elite tennis players, athlete mental health

## Abstract

**Introduction:**

This research determines mindfulness meditation promotes psychological recovery and lessen burnout amongst top-level tennis athletes. It also examines the modifying effect of psychological resilience on this association.

**Methods:**

The use of a quantitative cross- sectional design was used where 120 of the top and famous tennis players participated (mean age = 24.8 years). They were asked to fill in validated surveys that assessed mindfulness meditation, recovery, burnout, and psychological resilience. The analysis of data was carried out through structural equation modeling with SmartPLS 4 that was used in order to test direct, indirect, and interaction effects.

**Results:**

Structural equation modeling identified the relationship to be significant among the variables. The Mindfulness meditation has had a positive connection with recovery (*β* = 0.42, *p* < 0.001) and a negative relationship of burnout (*β* = –0.30, *p* < 0.001). The relationship between mindfulness meditation and the burnout was mediated by (partially) recovery (*β* = –0.19, *p* < 0.001). Mindfulness meditation had a significant effect on recovery (*β* = 0.42, *p* < 0.001) and psychological resilience moderated its effects (*β* = 0.22, *p* < 0.01), whereby more resilient athletes enjoyed more effects of mindfulness meditation. The model accounted to 41 percent variance in recovery and 53 percent in burnout.

**Discussion:**

The value of mindfulness to the recovery process is complemented by the element of psychological resilience, and this aspect should not be underestimated in terms of its significance in competitive sports settings.

## 1 Introduction

Elite tennis is a sport that is highly demanding for the players as they have to have a lot of physical, technical and psychological endurance if they want to do well. This means that a player not only has to participate in tough training sessions but also in frequent competitions and has to cope with high-pressure situations along the way which is the major cause of mental and physical exhaustion (Talebiazar et al., 2025). The recurring character of the matches, traveling, and the major emotional upheavals are major causes of chronic fatigue, emotional depletion, and cognitive overload (Kellmann and Kallus, 2024; Gustafsson et al., 2011). While the aspect of physical and recovery was known the one to be promoted mainly, however, the psyche dimension of recovery is now gaining much-needed attention in sports.

More and more people within the tennis community have expressed their concern about the growing number of athlete burnout cases. Athlete burnout is a psychological syndrome that points out to signs as emotional exhaustion, Lessened sense of professional accomplishment, and sport deterioration (Mahmoud, 2025). Burnout is not just a major obstacle preventing athletes from reaching their best potential but also it is something that eventually causes them to disengage completely and in longer time terms, it causes mental illnesses as well. The reminders of the research have picked up the facts and found that it is not only physical but also mental overload that is largely responsible for the issue, however, few measures of intervention singling out this target have been advanced by the researchers (Gerber et al., 2018; Smith et al., 2021).

While there are many recovery tools for the body, elite tennis players usually do not have proper guidance in the area of mental well-being. Various studies have found that mindfulness meditation which involves noticing your thoughts without judging, can help handle stress and improve emotions in people (Baer et al., 2006; Gardner and Moore, 2004; Kabat-Zinn, 2003). Even so, there is very little research on how it helps top tennis players, mainly for preventing fatigue by enhancing recovery. Because of this, research studies need to test the value of mindfulness in providing benefits to tennis players.

The goal of this research is to find out if mindfulness meditation is useful in decreasing burnout in elite tennis players by focusing on psychological recovery. It also looks at how being mentally strong or psychologically resilient, can boost the link between practicing mindfulness and the outcomes of the recovery. By using various approaches, scientists can easily determine when and for whom mindfulness works best.

This study seeks to answer the following questions:

1. Does mindfulness meditation improve recovery in elite tennis players?
2. Does improved recovery mediate the relationship between mindfulness meditation and reduced burnout?
3. Does psychological resilience moderate the relationship between mindfulness meditation and recovery?

The research introduces an approach that helps athletes by combining sports psychology models with mindfulness meditation drawing on the Conservation of Resources (COR) Theory (Hobfoll, 1989) and the Mindfulness-to-Meaning Theory (Garland et al., 2015). The model underlines that psychological recovery is important and that being resilient boosts performance, offering a better explanation of how elite athletes can keep performing well as time passes. Examples of such application are mindfulness-based intervention programs that help tennis professionals, coaches and sports psychologists lessen symptoms of burnout and build better mental health during competitions.

## 2 Literature review

### 2.1 Mindfulness meditation in sports

Mindfulness meditation involves paying close attention to your present moment and looking at your thoughts, feelings and body sensations from an impartial viewpoint (Fletcher and Sarkar, 2012; Galante et al., 2024). Mindfulness is understood in sports to increase attention, manage emotions well and keep performances consistent (Scheutzow et al., 2022). Paying attention to the present and not reacting to situations keep athletes in control under pressure, lessen brain distractions and help them recover fast if they make mistakes (Basque et al., 2021).

There is support from the research that mindfulness programs increase concentration, help suppress anxiety before competition, and make it easier for athletes to enter a flow state (Baltzell and Summers, 2017). Studies in basketball (Koop and Jooste, 2023), swimming (Scott-Hamilton et al., 2016), and archery have shown that mindfulness not only results in improved individual performance, but also provides advantages such as team cohesion and a psychologically safe training environment (Gardner and Moore, 2004).

### 2.2 Athletic recovery

Recovery covers the aspects of the body, mind and emotions. To recover from injuries, you need to rest, take proper nutrition and use physical therapy. Recovering cognitively means giving the mind a break and then focusing on tasks that make you feel less strain (Ballas et al., 2024; Ong et al., 2024). “Emotional recovery,” as well, is about balancing your feelings and lessening emotional tiredness (Ong et al., 2024). All of these exercises play a big part in keeping athletes fit and safe in elite sports. When the body does not recover properly, you can experience growing tiredness, poor thinking skills and mental health problems (Dyer et al., 2021). Thus, fully restoring an athlete’s health requires mindfulness meditation and other similar tools that assist with both emotional and intellectual recovery, especially in stressful situations as found in high-profile sports like tennis.

Psychological recovery involves processes to which the 113 depleted cognitive and emotional resource of a training or event due to stress, 114 competition or training has been restored. It involves psychological rest, emotional 115 stability, and the capacity to think apart of the sporting world pressures (Kenttä and Hassmén, 1998). Psychological resilience, meanwhile, is the ability to adapt effectively to a disaster, trauma, or other serious stressful event; the ability to remain healthy or recover despite stress (Fletcher and Sarkar, 2012).

### 2.3 Burnout in elite tennis

When stress from competition lasts for a long period and people do not relax enough, they can experience burnout. In the top levels of tennis, burnout is visible in tiredness, little success and a loss of sport value. Some reasons for burnout include having too much to train, traveling all the time, facing high performance targets and not having enough control over their personal decisions (Hammer, 2021). The outcomes are very damaging, including less effective work, retiring early and suffering from different mental problems such as anxiety and depression. Although many physical solutions are in use to prevent fatigue, psychological ways to help recover from it are still underdeveloped. Mindfulness helps tennis players manage the pressures of their sport which lessens the risk of emotional and physical exhaustion known as burnout.

### 2.4 Mediation and moderation in mindfulness research

Scientists in sport psychology have used mediation and moderation to figure out how mindfulness affects athletes. According to mediation models, the positive effects of mindfulness on lower burnout happen because of better recovery (Kirby et al., 2024). As a result, mindfulness improves an athlete’s recovery from stress and thoughts which helps them avoid burnout. Moderation deals with discovering how personal psychological features strengthen or weaken the effects of mindfulness (Hepburn et al., 2021). Strong-minded athletes might make better use of mindfulness to heal faster. For this reason, the study offers a two-part model in which recovery connects mindfulness and burnout, while resilience affects the connection between mindfulness and recovery in high-performance fields.

### 2.5 Theoretical framework

#### 2.5.1 Mindfulness-to-meaning theory

According to this theory, mindfulness encourages positive changes in how we think and feel. This means that mindfulness allows individuals to look at stressful events from a distance and handle their emotions better. If a person does not judge old experiences harshly, they can change how they react which leads to a more adaptive way of feeling. When it comes to sports, athletes can become more emotionally strong and recover more rapidly after facing difficulties (Galante et al., 2024). Mindfulness can make it easier for athletes to recover and reduce burnout because it helps them look at stress differently.

#### 2.5.2 Conservation of resources (COR) theory

According to Hobfoll’s Conservation of Resources Theory, anything that threatens somebody’s resources like energy, focus and inner peace can cause stress. When resources run out without being replenished, burnout takes place. In this sense, mindfulness meditation helps athletes to save their energy for important tasks (Salahuddin and Waheed, 2021). Thanks to psychological resilience, athletes have extra resources that help them deal with pressure and gain more from rest after training. Resource management for continuous performance is a goal they share which is why COR ties mindfulness and resilience together.

#### 2.5.3 Proposed conceptual model (for SmartPLS)

The literature and theory, the model in this case investigates how Mindfulness Meditation, an independent variable, is linked with Recovery, Burnout and Psychological Resilience. It suggests that mindful meditation can help tennis players heal faster, therefore cutting the risk of burnout (Brouwer et al., 2024). Moreover, it indicates that the connection between mindfulness and recovery for athletes depends on their ability to bounce back mentally. The PLS-SEM approach using SmartPLS is used to study direct, indirect and interaction effects in a single and clear model.

#### 2.5.4 Hypotheses development

With reference to current literature and theories, this work suggests a set of hypotheses to show how mindfulness meditation, recovery, burnout and psychological resilience are connected for elite tennis players.

*H1*: Mindfulness meditation is positively associated with recovery.

Mindfulness mediation teaches a person to be present and accept situations without judgment which may help them recover from stress (Smith et al., 2021). Since mental strength is always needed in elite sports, mindfulness can help athletes clear their minds, think straight and relax their bodies (Abdelmageed et al., 2024). Tests on this subject demonstrate that using mindfulness techniques regularly can help people sleep better, control performance pressure and manage their emotions during recovery (Wright et al., 2021). Hence, greater practice in mindfulness meditation is expected to contribute to a faster recovery for tennis players.

*H2*: Recovery is negatively associated with burnout.

Recovery serves to protect athletes from burnout since it lets them restore their mental, emotional and physical energy. If athletes do not rest enough, their built-up stress and tiredness make them more susceptible to having burnout symptoms, for example, emotional exhaustion, being cynical or losing motivation (Bégin et al., 2022). It is consistently proven in studies that athletes who take care of their recovery burn out less and perform well in the long run. It is therefore thought that tennis players who obtain more complete recovery is associated with reduced burnout.

*H3*: Mindfulness meditation is negatively associated with burnout.

Doing mindful meditation can help people handle stress and make sure their emotions are not drained. When athletes focus on the present and work on their thoughts, they find it easier to compete with pressure. Being able to regulate emotions goes against the signs of burnout (Rojas et al., 2023). Many research studies report that mindfulness training eased burnout in both professional and amateur athletes. For these reasons, it is believed that mindfulness meditation is connected to reduced burnout in elite tennis players.

*H4*: Recovery mediates the relationship between mindfulness meditation and burnout.

Although mindfulness cuts down stress and raises our feelings of well-being, its role in stronger recovery is highly significant, too. Mindfulness makes it easier to let go of emotions caused by difficulties which contributes to faster recovery in the mind (Caleshu et al., 2022). Because of this process, there is less likelihood of experiencing burnout. So, it seems that reducing burnout through mindfulness meditation happens mainly because it improves an athlete’s ability to recover (Reed et al., 2023). It is thought in this study that mediation explains the link between mindfulness meditation and burnout.

*H5*: Psychological resilience moderates the relationship between mindfulness meditation and recovery, such that the relationship is stronger at higher levels of resilience.

**Figure 1:**
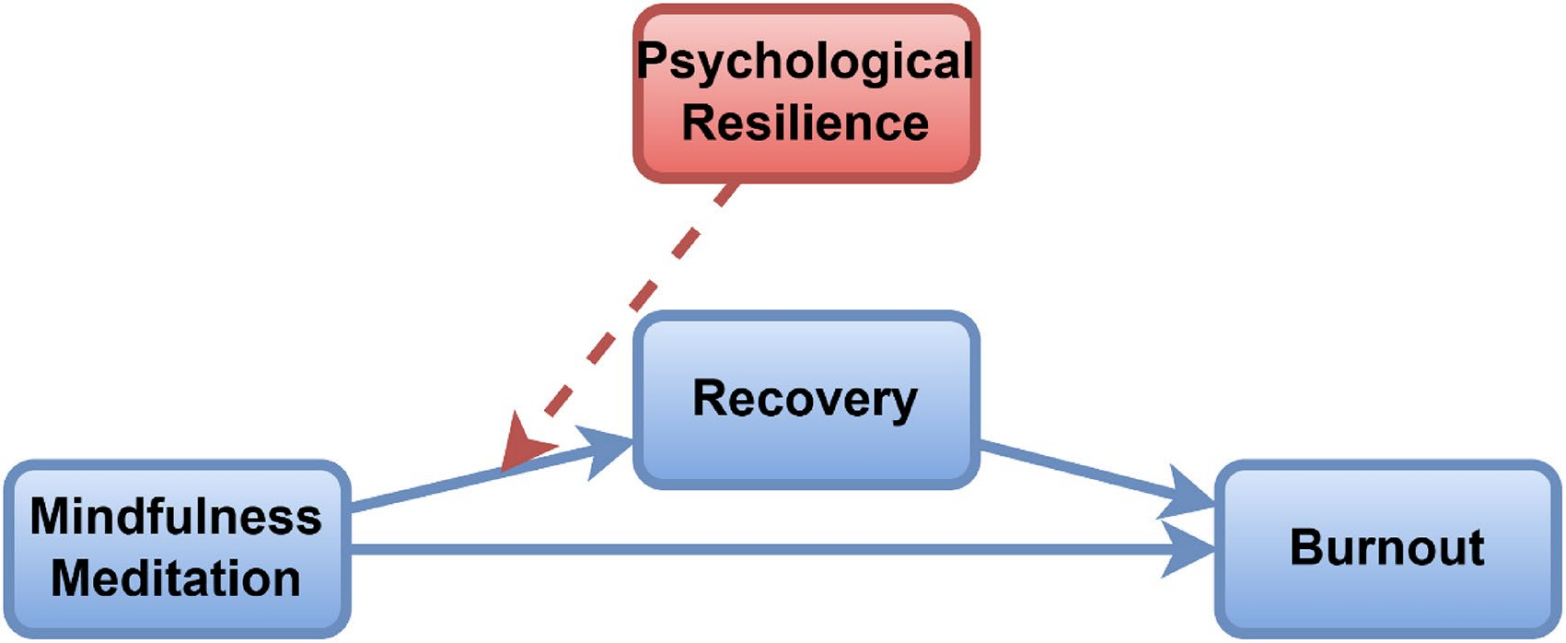
Hypothesis diagram.

The capacity to withstand adversity may make mindfulness meditation more effective. If someone is resilient, they are generally ready to practice mindfulness and see its benefits from their recovery (Homberg and Jagiellowicz, 2022). People with less resilience sometimes struggle to stay aware mindfully when things get stressful which may affect their overall gains from meditation (Polizzi et al., 2023). For this reason, resilience may improve the link between mindfulness and how quickly a person recovers. It is believed that the connection between mindfulness meditation and recovery is greatest for athletes who are psychologically strong as seen in Figure 1.

## 3 Methodology

### 3.1 Research design

The study is based on quantitative, cross-sectional data to look into how mindfulness meditation, recovery, burnout and psychological resilience relate in elite tennis players (Gasión et al., 2023). The analysis of data was accomplished through the SmartPLS 4 which is the software of variance-based structural equation model (SEM). SmartPLS was chosen due to its strengths of small sample sizes (*N* = 200 and below), non-normality of data, and measurement of the complex difference between mediation and moderation at the same time, which is best used in exploratory models in the psychology of sports (Hair et al., 2021).

### 3.2 Participants

The inclusion criteria stated that people had to be aged 18 or older, currently competing and ready to complete a survey. The elite tennis players that took part in the study were 120 (84 males and 36 females). They used to have a mean age of 24.8 years (SD = 3.6), and a competitive experience of 8.4 years (SD = 2.1) on average. At the time of data collection, all players were either engaged in national or international tournament.

### 3.3 Sampling method

Purposive sampling method was used to choose participants whose originating places were tennis academies, training camps and professional events. This approach implies a preconceived and theoretically grounded, selection of respondents with the best probability to gather rich and most relevant data, in this instance, of elite athletes, high stress levels, and most apt to respond to a mindfulness intervention (Palinkas et al., 2013). This outcome made our sample situational enough to explore the connection between mindfulness, recovery, and burnout.

### 3.4 Data collection

To assess the effect of the training sessions, participants completed one, self-report questionnaire in person (at the training camps or tournaments), or online (through the secure survey link), logistically convenient and the participant decided logistical into preference. The scores of all constructs followed the 5-point Likert scale (1 = Strongly Disagree, 5 = Strongly Agree). Factor loadings indicate the relationship between each measured (e.g., MM1) and its conceptual latent (e.g., Mindfulness Meditation). The loading above 0.65 shows that the item has a great convergence level, and this is a satisfactory measure of the construct. Questionnaire, where all entries are labeled (MM1, MM2, R1, R2, etc.) and a source scale is denoted in the Table 1. Table 1 Mindfulness meditation is the latent variable (Construct). Individual items (e.g., MM1) are seen as indicators of that construct. The Factor Loading (e.g., 0.72 of MM1) is a measure of the extent to which that item measures the construct—it is said that the loading should be above 0.65. In the Five facet mindfulness framework, item MM1 (Observes bodily sensations with awareness) is the same as the Observing aspect and MM4 (Accepts experiences without judgment) the same as the Non-judging aspect (Baer et al., 2006). Burnout was measured by means of providing Athlete Burnout Questionnaire (ABQ) (Gerber et al., 2018). According to previous validation research, item B1 (Feels emotionally drained as a result of the training requirements), loaded highest (0.76), and is associated with the subscale of Emotional Exhaustion. This Table 1 comes from the measurements and findings taken from the study’s measurement and structural models.

**Table 1:** Construct, statement and factor loading.

Construct	Item	Statement	Factor loading	References
Mindfulness meditation	MM1	Observes bodily sensations with awareness	0.72	Five Facet Mindfulness Questionnaire (FFMQ)—Non-judging facet (Baer et al., 2006)
	MM2	Describes feelings without over-identifying with them	0.68	
	MM3	Acts with awareness rather than on autopilot	0.75	
	MM4	Accepts experiences without judgment	0.71	
Recovery	R1	Feels mentally relaxed after rest periods	0.74	Recovery-Stress Questionnaire for Athletes (RESTQ-Sport) (Kellmann and Kallus, 2024)
	R2	Regains emotional balance after intense matches	0.70	
	R3	Replenishes physical energy effectively	0.69	
	R4	Returns to optimal focus quickly after stress	0.73	
Burnout	B1	Feels emotionally drained from training demands	0.76	Athlete Burnout Questionnaire (ABQ) (Gerber et al., 2018)
	B2	Doubts personal accomplishment in tennis	0.72	
	B3	Loses interest or passion for playing tennis	0.68	
	B4	Feels detached from tennis due to overtraining	0.70	
Psychological resilience	PR1	Bounces back quickly after setbacks	0.78	Fletcher and Sarkar (2012) -*Conceptual foundation for resilience in sport*
	PR2	Remains calm under pressure	0.74	
	PR3	Maintains confidence despite challenges	0.71	
	PR4	Stays focused and composed during adversity	0.75	

This study relied on a full measurement model and analysed the factor loadings and the key aspects of four focal areas: Mindfulness Meditation, Recovery, Burnout and Psychological Resilience. All constructs were measured using existing scales and the items for every construct had strong factor loadings (over 0.65), proving that they matched well with their latent factors. For example, “Observes bodily sensations with awareness” in Mindfulness Meditation (0.72) went toward Focused Awareness and how “Accepts experiences without judgment” (0.71) was consistent with Acceptance, just as in the Five Facet Mindfulness Questionnaire (FFMQ). The Recovery construct was also shown to have substantial loadings on cognitive and emotional areas by the RESTQ-Sport scale such as “Returns to optimal focus quickly after stress” (0.73). ABQ scores for the Burnout construct showed that “Feels emotionally drained from training demands” was the strongest item, falling under Emotional Exhaustion which is a main aspect of athlete burnout. Finally, Psychological Resilience scored very well, especially for the item “Bounces back quickly after setbacks” (0.78) in the Adaptability group, indicating that resilience is an adaptable trait that strengthens mental toughness and allows elite players to maintain good performance over time.

### 3.5 Analysis tool

SmartPLS 4 was used to analyze the data since it helps in both predictive modeling and checking theories. It was selected for being able to manage medium and large models, even if the sample size is quite small or the data follows a non-normal distribution.

## 4 Results

### 4.1 Descriptive statistics

Table 2 present an overview of who the participants are and their average scores on the examined constructs. The 120 respondents—composed of 70% men and 30% women—had an average competition experience of 8.43 years (SD = 2.1). The study’s results noted that participants had moderate mindfulness (3.8), average recovery (4.1), moderate burnout (2.9) and good psychological resilience (4.0).

**Table 2:** Descriptive statistics.

Construct	Mean	SD	Min	Max
Mindfulness meditation	3.80	0.52	2.4	4.9
Recovery	4.10	0.58	2.7	5.6
Burnout	2.90	0.65	1.5	4.7
Psychological resilience	4.00	0.48	2.8	5.0

### 4.2 Measurement model assessment

In order to check how well the measurement model performed, SmartPLS 4 was used to test its reliability and validity. Table 3 shows all the constructs were internally reliable, as Cronbach’s Alpha and Composite Reliability (CR) were more than 0.70 for every construct. Convergent validity was supported because all Average Variance Extracted (AVE) values more than 0.50 indicated that all latent variables were properly measured.

**Table 3:** Reliability and validity.

Construct	Cronbach’s alpha	CR	AVE
Mindfulness meditation	0.88	0.91	0.56
Recovery	0.86	0.90	0.59
Burnout	0.83	0.88	0.52
Psychological resilience	0.89	0.91	0.60

By using the Heterotrait-Monotrait ratio (HTMT) criterion, it was established that the HTMT values were all lower than the 0.85 threshold, proving that the constructs are different from each other.

### 4.3 Structural model assessment

Researchers measured the structural model by looking at path coefficients, *R*^2^, *f*^2^ and *Q*^2^. The significance of the main effects was checked using the bootstrapping method with 5,000 resamples. The values of 0.41 for Recovery and 0.53 for Burnout point to some useful explanations about the variables. In addition, *Q*^2^ scores were positive which indicates that the model has some predictive ability.

Table 4 shows the findings for all hypotheses. Every pathway—from stimuli to responses—played its part, and both mediation and moderation effects were statistically confirmed. The findings have been in favor of all the five hypotheses (H1–H5): (H1) That mindfulness meditation affected recovery (H2) that recovery affected burnout (H3) that mindfulness directly affected burnout (H4) that psychological resilience moderated relationship (H5).

**Table 4:** Hypotheses testing.

Hypothesis	Path	*β*	*t*-value	*p*-value	Result
H1	Mindfulness → Recovery	0.42	6.25	<0.001	Supported
H2	Recovery → Burnout	−0.45	7.02	<0.001	Supported
H3	Mindfulness → Burnout	−0.30	4.56	<0.001	Supported
H4	Mindfulness → Recovery → Burnout	−0.19	4.01	<0.001	Mediation supported
H5	Mindfulness × Resilience → Recovery	0.22	3.45	<0.001	Moderation supported

**Figure 2:**
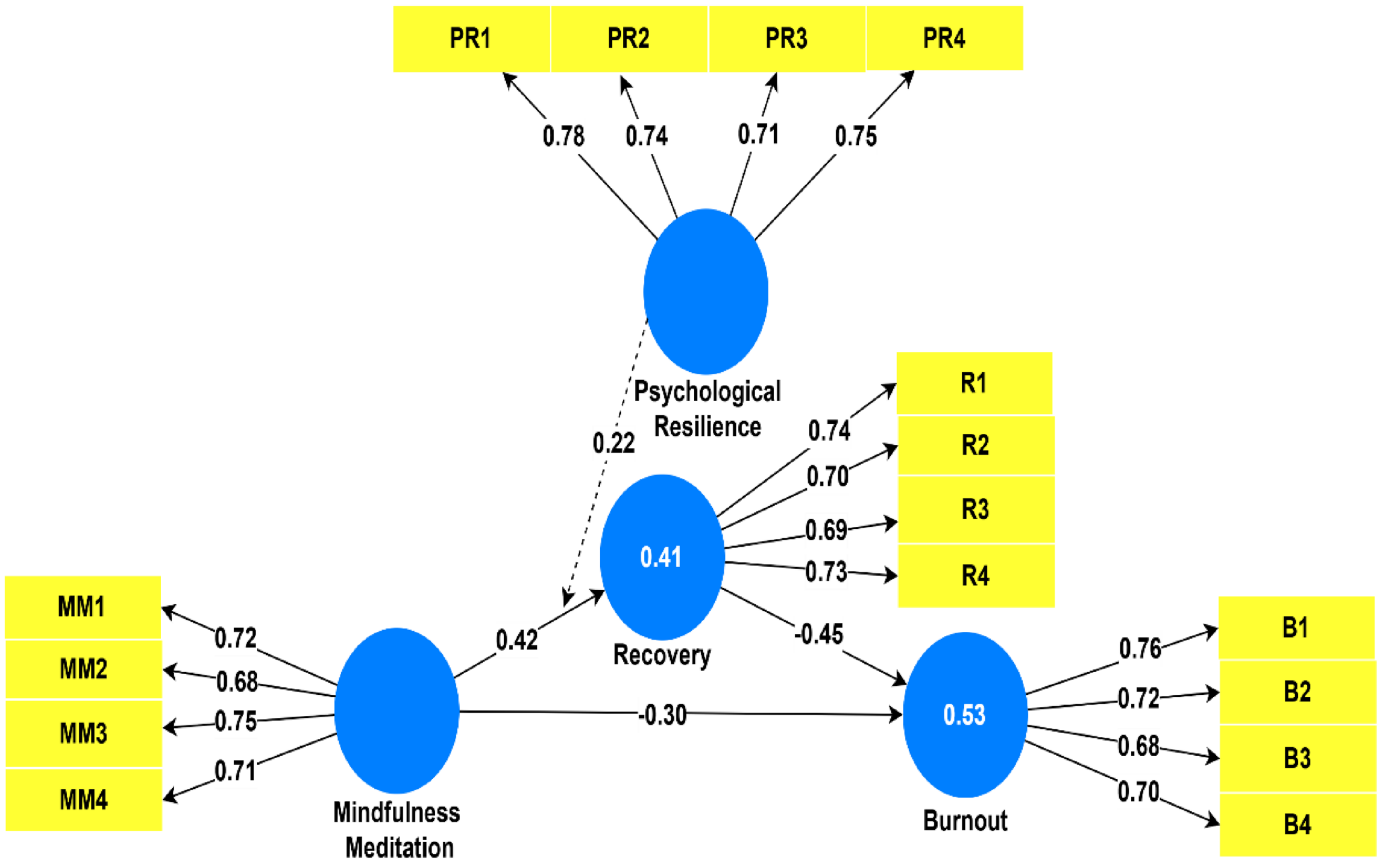
Structural model with path coefficients and *R*^2^ values.

According to the results, multiple important relationships is identified in the model. There was a significant positive relation (*β* = 0.42, *t* = 6.25, *p* < 0.001) between higher mindfulness and greater Recovery. This means that the more recovery people experience, the less likely they are to feel burnout (*β* = −0.45, *t* = 7.02, *p* < 0.001). In addition, Burnout levels were lower when Mindfulness was practiced (*β* = −0.30, *t* = 4.56, *p* < 0.001). Recovery plays a mediating role and lessens Burnout because of a significant indirect impact of Mindfulness (*β* = −0.19, *t* = 4.01, *p* < 0.001). High levels of Resilience seem to make the link between mindfulness and recovery stronger (*β* = 0.22, *t* = 3.45, *p* < 0.01) than it is for individuals with less resilience as seen in Figure 2.

### 4.4 Mediation and moderation effects

There was a significant effect of mindfulness on burnout because of recovery (*β* = −0.19, *p* < 0.001) which means partial mediation happened. Higher resilience appears to make the impacts of mindfulness more important for athletes (*β* = 0.22, *p* < 0.01).

The Table 5 indicates model did well at predicting Recovery and Burnout for *R*-Squared of 0.41 and 0.53 respectively, demonstrates that the model has explained 41 and 53 percent of the variation in the outcome in Recovery and Burnout, respectively. Indicatively, the model variables (mindfulness, resilience and their interaction) explain more than half of the variation found in the levels of burnout among sportspeople—which is indicative of high predictive power. It can be seen from the effect size measures (*f*^2^) that factors predicted about 18% of Recovery and about 23% of Burnout. Both the Recovery and Burnout predictive relevance (*Q*^2^) numbers are greater than zero which proves that the model can successfully forecast the values of both measures.

**Table 5:** *R*^2^, *f*^2^, and *Q*^2^.

Construct	*R* ^2^	*f* ^2^	*Q* ^2^
Recovery	0.41	0.18	0.27
Burnout	0.53	0.23	0.32

The results proved the original hypothesis and indicated that mindfulness meditation helps reduce burnout, as well as increasing the pace of recovery. The positive effects of mindfulness on recovery are larger for athletes who are mentally tough.

## 5 Discussion

### 5.1 Interpretation of key findings

It was found through this study that using mindfulness meditation substantially supports the recovery of elite tennis players and that this, in turn, lowers the effects of burnout (Miyoshi et al., 2022). Besides helping people recover from a burnout, mindfulness is shown to reduce psychological exhaustion both directly and indirectly by promoting recovery (Cohen et al., 2023). It was noticed that strong psychological resilience boosts the beneficial effect of mindfulness on how fast people recover (Muir et al., 2022). Therefore, people with a strong sense of resilience may make better use of mindfulness to recover from injuries.

### 5.2 Theoretical implications

These researches also add to and broaden the scope of two theoretical models. For one, the Mindfulness-to-Meaning Theory is reinforced by the fact that mindfulness enables athletes to see stressful events in a positive and accepting light, resulting in better emotional and cognitive recovery. Furthermore, it also supports Conservation of Resources (COR) Theory by claiming that the presence of recovery and resilience can be deemed as the resources available to prevent burnout. The interaction between recovery (as a mediator) and resilience (as a moderator) in this study offers a more resource-based critique of how mindfulness maintains athlete well-being/performance—consistent with the Conservation of Resources Theory.

### 5.3 Practical implications for athlete mental health and coaching strategies

As shown by Robin et al. (2025), mindfulness intercessions not only lower burnout, but also improve self-efficacy and on court performance—adds another reason why they should be incorporated into elite tennis training programs. There is much that coaches, sport psychologists, and athletic trainers can do on a regular basis to develop mindfulness which enables them to stay focused, be more relaxed, and recover more quickly after matches or intensive workouts. In view of the moderating role of resilience, resilience-building strategies—like cognitive reframing or stress inoculation training—could further heighten the beneficial effects of mindfulness. In particular, institutions and sports academies should make clear that psychological recovery is just as important as physical recovery in the prevention of burnout and the sustenance of peak performance over a period of time.

### 5.4 Limitations and future research implications

However, the research also has certain limitations. For instance, the cross-sectional design of the study precludes inferences about cause and effect. In the future, researchers could use longitudinal or experimental designs to follow the developments over time for a more effectual identification of the reasons for such developments. In the second place, the research was exclusively concentrated on the elite tennis players thereby making the findings not applicable for other disciplines or non-elite populations. Additionally, although self-reported scales were used for the measurement, using physiological or behavioral indicators of recovery and burnout might allow for more objective insights in the next studies. The last limitation is that certain other conceptual mediating mechanisms (e.g., emotional regulation) or situational moderators (e.g., coach support) can deepen the insights into how mindfulness functions in the case of high-performance sports.

## 6 Conclusion

This study investigated the functioning of mindfulness meditation in improving recovery and sparing burnout among professional tennis players, where the main points were the medium of recovery and the agent impact of the mood of the person. The results clarified that the practice of meditation has a positive impact on the psychological state of the player and lowers the risk of burnout. The process of recovery, in addition to being a mediator, was also illustrated as the important cause in that it can facilitate the functions of the mental aspect of mindfulness and decrease the level of burnout symptoms. The flexing of the resilience was complementary in disclosing that the application of mindfulness by the athletes is efficient only in cases where the athletes had a progress in the ability to adapt and be resistant to pressure.

The study has several contributions to the existing literature. The conceptually, this work joins the Mindfulness-to-Meaning Theory and Conservation of Resources (COR) Theory in the field of elite sports, hence, coming up with a powerful model that describes the cooperation between mindfulness and psychological resilience to save athletes’ mental resources. Furthermore, the study pioneers the application of SmartPLS to confirm mediation and moderation in a structural equation model, thereby, it promotes a new method of analysis for the investigation of the mental factor in sports. From the standpoint of usefulness, it supplies sport psychologists and coaches with tested guidelines and practices for working with mindfulness and resilience training in such a way as it will assist athletes in protecting from burnout and in nurturing the sustainability of good performance.

In short, the present study has revealed that mindfulness of high performance in tennis players can be both, a promoter of recovery, and a protector against burnout throughout the research. By bringing psychological recovery strategies into the training process, coaches and other stakeholders have a chance to guarantee the endurance not only of the body but also of the mind for their top athletes.

## Data Availability

The raw data supporting the conclusions of this article will be made available by the author without undue reservation.
